# Acaricidal Potential and Ecotoxicity of Metallic Nano-Pesticides Used against the Major Life Stages of *Hyalomma* Ticks

**DOI:** 10.3390/life12070977

**Published:** 2022-06-29

**Authors:** Tean Zaheer, Mahmoud Kandeel, Rao Zahid Abbas, Shanza Rauf Khan, Tauseef ur Rehman, Amjad Islam Aqib

**Affiliations:** 1 Department of Parasitology, University of Agriculture, Faisalabad 38040, Pakistan; rao.zahid@uaf.edu.pk; 2 Department of Biomedical Sciences, College of Veterinary Medicine, King Faisal University, Al-Hofuf 31982, Saudi Arabia; 3 Department of Pharmacology, Faculty of Veterinary Medicine, Kafrelshikh University, Kafrelshikh 33516, Egypt; 4 Department of Chemistry, University of Agriculture, Faisalabad 38040, Pakistan; shanza.khan@uaf.edu.pk; 5 Department of Parasitology, The Islamia University Bahawalpur, Bahawalpur 63100, Pakistan; drtauseef@iub.edu.pk; 6 Department of Medicine, Cholistan University of Veterinary and Animal Sciences, Bahawalpur 63100, Pakistan; amjadislamaqib@cuvas.edu.pk

**Keywords:** *Hyalomma*, ZnO, MgO, Fe_2_O_3_, nanoparticles, tick bioassay, ecotoxicity, snails

## Abstract

Ticks (Acari: Ixodidae) are blood-feeding parasites capable of transmitting diseases to animals (Piroplasmosis) and humans (Congo fever and Lyme disease). The non-judicious use of chemical acaricides has led to the development of acaricide-resistant ticks, making the control of ticks and tick-borne diseases difficult. This study reports the efficacy of magnesium oxide (MgO), iron oxide (Fe_2_O_3_), and zinc oxide (ZnO) nanoparticles (NPs) as alternatives to traditional acaricides/pesticides using in vitro tests against major representative stages of *Hyalomma* ticks. Nanopesticides were chemically synthesized as rods (Fe_2_O_3_), stars (ZnO), and spheres (MgO) and were characterized by XRD and SEM analysis. The in vitro bioassays included adult immersion, larval immersion, and egg immersion tests. Non-target effects of the nanopesticides were evaluated using snails. The LC_50_ values of Fe_2_O_3_ NPs (4.21, 2.83, and 0.89 mg/L for the tick adult, larval and egg stages, respectively) were lowest, followed by those of MgO (4.27, 2.91, and 0.93 mg/L) and ZnO (4.49, 3.05, and 0.96 mg/L). Fe_2_O_3_ NPs were capable of arresting oviposition and larval hatching in the studied ticks in vitro. The snail toxicity experiments revealed minimum to mild off-target effects for all tested nanopesticides. This study is the first to report on the comparative efficacy of magnesium, iron, and zinc nanomaterials for toxicity in egg, adult and larval stages of *Hyalomma* ticks. Further studies establishing the efficacy of NPs against ticks, as well as their safety at host–human environment interface could lead to promising nanopesticide applications.

## 1. Introduction

Ticks are blood-feeding arthropods known to transmit lumpy skin disease, Q fever, rickettsiosis, ehrlichiosis, Boutonneuse fever and Lyme disease [1,2]. Livestock species in developing countries such as Pakistan face threats from multiple tick-related diseases [3,4], and estimated economic losses in Brazil and Mexico due to tick epidemics, as well as prevention and treatment, range from $573.61 million to $3.24 billion annually [5]. Although the transmission of these diseases affects livestock and threatens caretakers and pet animals [6,7], the widespread use of conventional therapies has led to drug resistance.

*Hyalomma* ticks represent one of the most significant disease-transmitting genera of Ixodidae due to their vector potential for livestock and impact on public health [8]. A growing tide of acaricidal resistance in ticks, combined with the slow development of chemical acaricide design and implementation, reveals the need for alternative options. Alternative control measures, either alone or in combination with chemical acaricides, constitute an integrated vector management approach shown to be effective in controlling ticks and tick-borne diseases [9]. To this end, the use of management strategies including rotational grazing, spelt pastures, alternative grazing, improved floor design, and the use of footbaths have been applied in combination with other methods for moderately effective tick control [10].

Metallic and non-metallic nanoparticles (NPs) have shown great promise for rapidly inducing toxicity and reducing lethal concentrations at various stages of the parasite’s life cycle [11]. NPs have been found to induce toxicity against some endoparasites and ectoparasites [12,13,14]. Magnesium, zinc, and iron, among other metals, are known to regulate cellular mechanisms in arthropods. MgO NPs have shown promising lethality via cell membrane permeability, nervous conduction and excitability, and intermediary metabolism. Similarly, zinc is crucial to DNA synthesis, mitosis, and cell proliferation, in addition to acting as an intracellular antioxidant in arthropods [15]. Iron is crucial in preventing oxidative stress within arthropods [16]. Given the pivotal roles of these metals in arthropod physiology, the current study evaluates the comparative acaricidal activity of ZnO, MgO, and Fe_2_O_3_ NPs against the major stages of *Hyalomma* ticks and determines their toxicity in snails (being one of suitable candidates for pesticide and ecotoxicology testing).

## 2. Materials and Methods

### 2.1. NP Synthesis and Characterization

Magnesium chloride (MgCl_2_·6H_2_O), sodium dodecyl sulfate (SDS), sodium hydroxide (NaOH), zinc acetate dihydrate (Zn(CH_3_COO)_2_·2H_2_O), polyethylene glycol (PEG), urea (NH_2_CONH_2_), iron chloride tetrahydrate (FeCl_2_·4H_2_O) and ammonia were purchased from Sigma-Aldrich USA.

**Synthesis of MgO NPs:** MgO NPs were prepared hydrothermally in the presence of surfactant [17]. The MgCl_2_·6H_2_O solution was prepared by dissolving 4 g MgCl_2_·6H_2_O in 40 mL distilled water. Four grams of SDS was added to the MgCl_2_ solution with constant magnetic stirring for 4 h at room temperature. Twenty mL of 2.5 M NaOH was added into the reaction mixture dropwise to maintain a pH of 12. The resulting white suspension was transferred into a stainless steel-lined solvothermal autoclave reactor and heated at 140 °C for 6 h. The obtained white precipitates were washed with distilled water and collected by centrifugation. The precipitates were dried at 60 °C for 24 h in a thermoelectric oven, ground, and then calcinated at 450 °C for 3 h.

**Synthesis of ZnO NPs:** Three grams of Zn(CH_3_COO)_2_·2H_2_O and 1 g urea were dissolved in 65 mL distilled water to prepare solution A. One gram of urea and 3 g PEG were dissolved in 65 mL distilled water to prepare solution B. Solutions A and B were mixed, and 13 mL of concentrated ammonia was added dropwise to maintain a pH of 12. The solution was poured into a Teflon vessel and heated in an autoclave reactor at 110 °C for 5 h. The product was washed, dried, ground to a fine powder, and calcinated at 550 °C for 5 h.

**Synthesis of Fe_2_O_3_ NPs:** Two grams of FeCl_2·_4H_2_O was dissolved in 6 mL distilled water, and 42 mL of concentrated ammonia was added. The reaction mixture was heated in an autoclave at 140 °C for 3 h. The product was washed, dried, and ground to a fine powder before application.

**NP characterization:** A Rigaku D/max Ultima III X-ray powder diffractometer operated at 40 kV and 0.130 A current and equipped with a Cu-Kα radiation source (wavelength of 0.15406 nm) was used for the XRD analysis of NP products. An SEM Quanta 250 operated at 30 kV was used to scan the NPs.

### 2.2. Tick Collection and Identification

Engorged ticks were collected from the ears, eyelids, lips, and tails of bovines (cattle and buffalo) with no reported history of acaricidal exposure within the previous 30 days [18]. Ticks were collected from the animals in untreated vials, irrespective of animal gender, age, and species, and were identified using a stereomicroscope (IM-SZ-210, Irmeco, Lütjensee, Germany) following an identification key developed by Walker et al. [19]. Tick anatomical features of the mouth, basis capitulum, coxa, and scutum, as well as other features, were assessed for identification at the genus level.

### 2.3. Tick Bioassays

We evaluated the efficacy of various concentrations of NPs against egg, larval and adult stages of collected *Hyalomma* ticks using the following bioassays: egg immersion (EIT), larval immersion (LIT) and adult immersion (AIT). All tick rearing and bioassays were performed within a biological oxygen demand incubator at a temperature of 28–30 °C and 80–90% relative humidity for the required time [20,21]. Briefly, partially fed and fully engorged female ticks were moved to separate tubes for incubation and subsequent ovipositioning [22]. After 11–35 days, females had laid eggs, and the shriveled and dead females were separated from the eggs to avoid microbial contamination. The eggs were then incubated until larval hatching. Mortality data was subject to the formula proposed in [23].

### 2.4. Contact Toxicity in Garden Snails

For the toxicity evaluation, 120 apparently healthy and active land snails were collected, regardless of gender, from the organic garden at the University of Agriculture, Faisalabad, Pakistan, where chemicals and pesticides had not been used in the previous 30 days. There were seven groups of snails, including two control (negative and positive) and five treatment groups. The seven groups were designated as follows: Group 1 (cypermethrin), Group 2 (deltamethrin), Group 3 (MgO NPs), Group 4 (ZnO NPs), Group 5 (Fe_2_O_3_ NPs), Group 6 (positive control, DMSO) and Group 7 (negative control, distilled water). The five treatment groups (all groups except controls) were further divided into four sub-groups receiving the following NP concentrations, with *n* = 5 snails randomly assigned to each subgroup: 0.01 mg/mL, 0.1 mg/mL, 1 mg/mL, and 10 mg/mL. A 50 µL of solution from each of a 0.01 mg/mL, 0.1 mg/mL, 1 mg/mL, and 10 mg/mL NPs solution was poured onto the anterior mouth side of each snail using the method described by Radwan et al. [24] with some modifications. The snails were transferred to Petri plates instead of plastic bottles to improve aeration. The top of each plate was covered with organza netting secured with rubber bands. The final dose received by each snail was 0.5 µg, 5 µg, 50 µg, and 500  µg for the four NP concentration subgroups within each treatment group. Snails were kept off feed for 5 days, and dead snails were analyzed by histopathology. The percentage of mortality on the 1st, 3rd, and 5th days was calculated based on the number of dead snails divided by the total number of snails tested.

**Applied formulae**:

$$Corrected\;Mortality=\frac{\%\;treated\;mortality-\%\;control\;mortality\;}{100-\%\;control\;mortality}\times 100$$

$$\%Mortality\;of\;snails\;at\;“x”\;time\;interval=\frac{Number\;of\;snails\;died\;at\;“x”time\;interval}{Total\;number\;of\;snails\;used\;in\;the\;trial}\times 100$$

x = 1st day (24 h); 3rd day (48 h); and 5th day (120 h)

**Histopathology**: The digestive glands of the snails were processed for dissection and fixation using Bouin’s fluid. Fixed sections were de-paraffinized in 5 µm thick sections, then hydrated and stained in hematoxylin for fifteen minutes. After washing with water, sections were stained with 1% eosin solution for two minutes. The samples were dehydrated with alcohol, cleared in xylene, mounted on clean slides using Canada balsam and covered with thin coverslips [25]. Salient pathological indicators included cell disintegration, bizarre nuclei ranging from karyolysis to severe karyorrhexis, complete pyknosis, numerous vacuolations, frequent dark granulation, and hemocyte infiltration.

### 2.5. Statistical Analysis

The LC_50_ and LC_99_ values and their associated confidence intervals were estimated from 24 h mortality/survivability data using Probit analysis by SPSS (SPSS for Windows, version 22; IBM, Armonk, NY, USA), with probability ≤0.05 considered significant.

## 3. Results

### 3.1. XRD Analyses of MgO, ZnO, and Fe_2_O_3_ NPs

**Figure 1:**
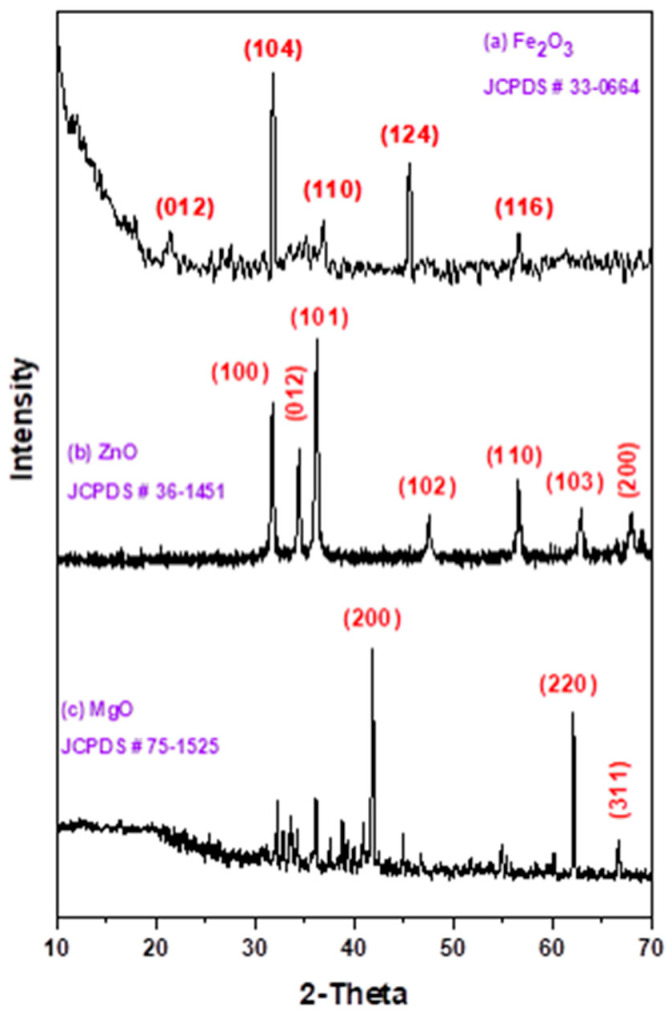
XRD pattern of synthesized (**a**) Fe_2_O_3_, (**b**) ZnO and (**c**) MgO nanoparticles. Intensity has been denoted in ‘a.u’ units.

XRD patterns of the synthesized products were plotted as 2 theta (θ) values vs. intensity (Figure 1). Miller indices of Fe_2_O_3_ for diffraction peaks at 2 theta (θ) 22°, 33°, 35°, 46°, and 56° were (012), (104), (110), (124), and (116), respectively (JCPDS # 33-0664) [26]. The set of 2 theta (θ) values and corresponding indices indicate that the synthesized product was Fe_2_O_3_ NPs. The XRD pattern showed that the peaks were not sharp, indicating that the product was not completely crystalline. Figure 1b shows the diffraction peaks at 2 theta (θ) 32°, 34°, 36°, 45°, 56°, 62°, and 68° with Miller indices of (100), (012), (101), (102), (110), (103), and (200), respectively, confirming that the synthesized product was ZnO NPs (JCPDS # 36-1451) [27]. For ZnO, the peaks were sharp, indicating a crystalline ZnO product. Figure 1c shows diffraction peaks at 2 theta (θ) 42°, 62°, and 66°, corresponding to (200), (220), and (311) Miller indices, respectively (MgO, JCPDS # 75-1525) [28]. All peaks were sharp, indicating that the synthesized product was crystalline. Overall, the ZnO NPs were purer and provided the sharpest peaks among all the synthesized products.

### 3.2. SEM Analyses of MgO, ZnO, and Fe_2_O_3_ NPs

**Figure 2:**
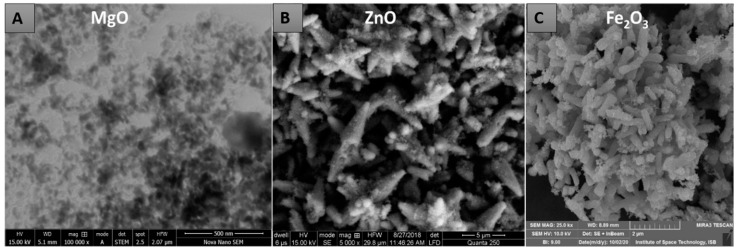
SEM images of nanoparticles: (**A**) MgO, (**B**) ZnO and (**C**) Fe_2_O_3_.

An SEM image of MgO NPs synthesized hydrothermally is provided in Figure 2. The product comprises fully dispersed spherical/oval-shaped NPs. Aggregation was not observed in this image. MgO NPs were 30–80 nm in size. A contrast was not observed, indicating that the particles were compact and not hollow. An SEM image of the ZnO NPs is shown in Figure 2. Polygonal, randomly oriented, unfused, star-like particles were observed. The boundaries of particles were clear, and every particle consisted of many spikes joined at the center. The spike lengths were not equal. The size of every star-like particle was approximately 4–8 µm, and the terminal ends of spikes were blunt rather than pointed. Urea acted as the directing morphology template; primary particles were formed initially and stabilized by urea molecules. Due to calcination, urea molecules decomposed into carbon dioxide and ammonia, forming star-like particles. The rod-shaped NPs of Fe_2_O_3_ are shown in Figure 2. These rod-shaped particles were randomly aligned, approximately 2 μm long and 100 nm wide. The terminal ends of rods were not pointed. The rods appeared to have emerged from spherical NPs because few spherically shaped minute particles adhered to the surface of the rods. As the rods were not aggregated, their surface was available for many interactions with the environment.

### 3.3. Tick Bioassays

**Figure 3:**
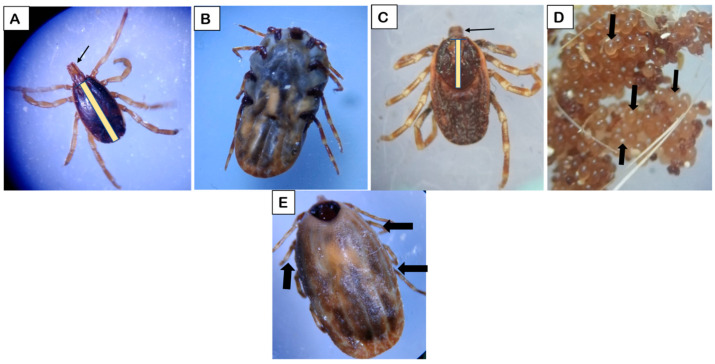
Stereomicrographs of *Hyalomma*. (**A**) male adult–yellow bar: scutum that covers most of the body; arrow: long mouth parts. (**B**) Partially fed female—ventral side showing a smaller degree of engorgement and an increase in size due to blood feeding. (**C**) Un fed female–yellow bar: scutum that covers only 1/3rd of the body; arrow: long mouth part. (**D**) Eggs—egg mass: black arrows indicate freshly laid eggs with a bright appearance. (**E**) Female adult tick showing dorsal view. Black arrows indicate the identification point of *Hyalomma* tick- pale rings on legs.

**Figure 4:**
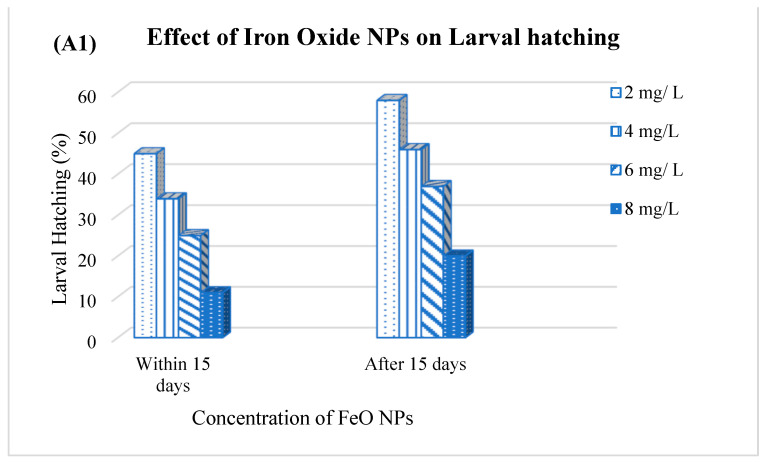

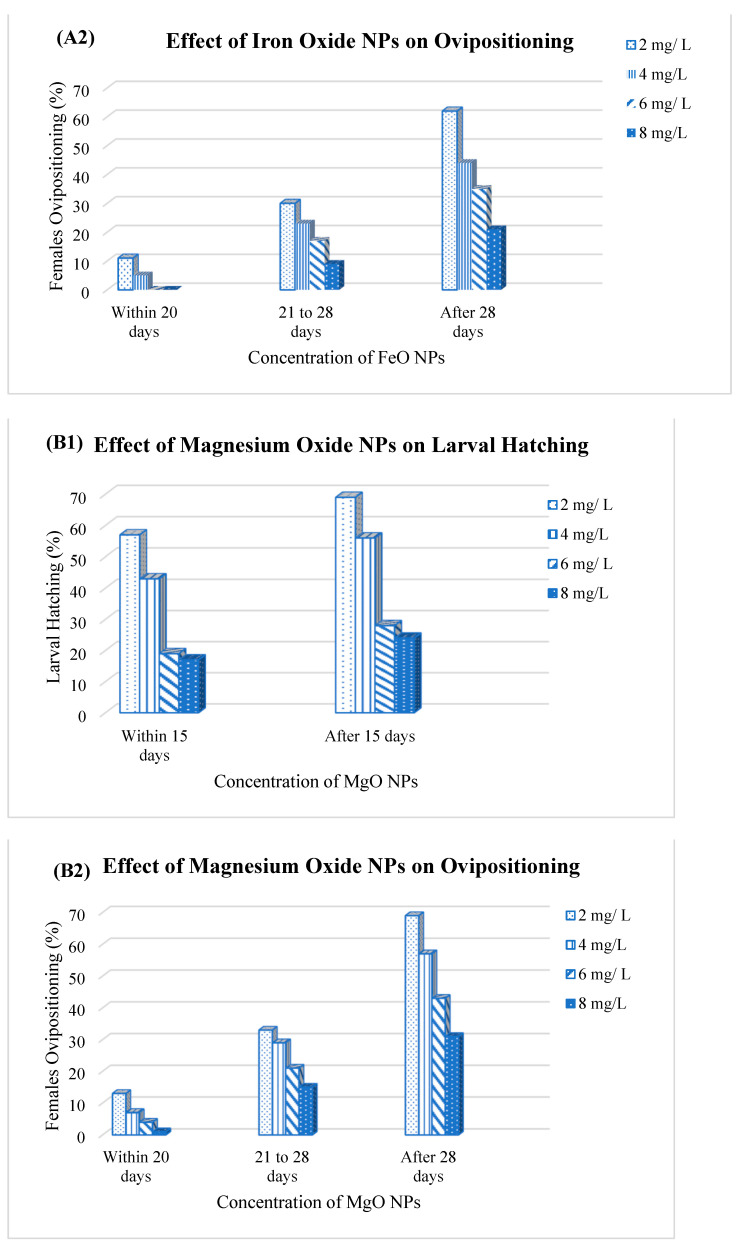

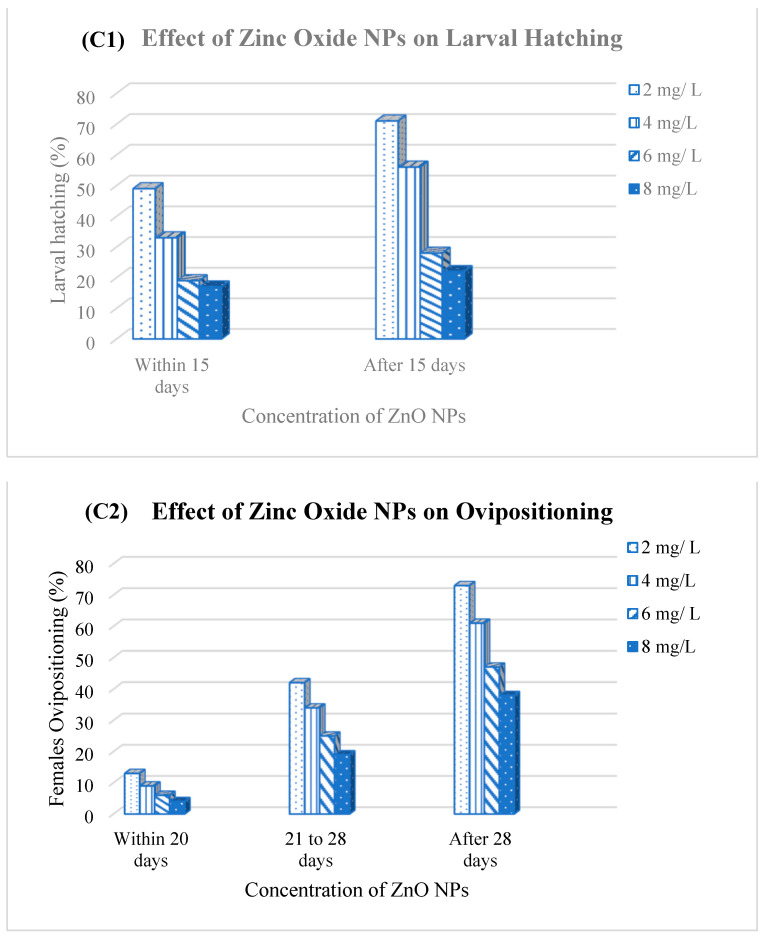
Percentage of larval hatching and adult female oviposition of ticks under the effect of iron oxide (**A1**,**A2**), magnesium oxide (**B1**,**B2**) and zinc oxide nanopesticides (**C1**,**C2**). NPs = nanopesticides/nanoparticles.

**Figure 5:**
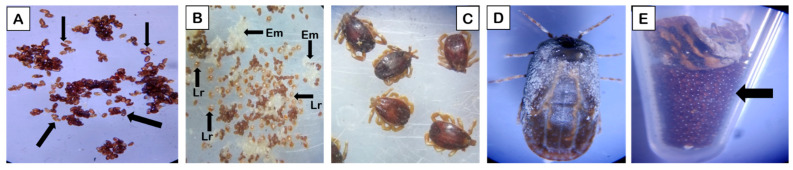
*Hyalomma* ticks subject to nanopesticides. (**A**) Desiccated egg mass with no larval hatching. (**B**) Larval hatching from eggs: small larvae (brown-colored, labelled as ‘Lr’) crawling around white egg masses (labelled as ‘Em’) are visible. (**C**) Dead male ticks. (**D**) Dead female without ovipositioning (dark-colored); no egg mass found, as in control group. (**E**) Ovipositioning in control group, with bright egg masses visible (arrow).

The preliminary stereomicrographs showing adult, larval and egg stages of Hyalomma ticks are shown in Figure 3. Iron oxide NPs demonstrated superior acaricidal activity against ovipositioning, larval emergence, and egg hatching relative to MgO and ZnO NPs, and ZnO NPs showed the least activity. Similarly, adult and larval mortality, regardless of NP concentration, was higher in ticks exposed to Fe_2_O_3_ NPs (Table 1). Adult female ovipositioning is expressed in percentages, divided into three major categories: egg laying within 20 days, egg laying from 21–28 days and egg laying after 28 days (Figure 4). The egg mass from which no larvae emerged, even after 35 days post treatment, was considered dead due to the NP treatment. The color of egg masses that desiccated without larval emergence changed to dark brown/black (Figure 5a). The egg-laying of Fe_2_O_3_ NP treatment groups was 62%, and the lowest lethal Fe_2_O_3_ NP concentration required to arrest larval hatching was LC_90_ = 1.69 mg/L. Mortality data, in terms of LC50 and LC90, along with associated confidence intervals, are mentioned in Table 1.

### 3.4. Ecotoxicity Analysis

As a preliminary investigation, all three NPs demonstrated minimum to mild off-target effects on the snails (Table 2). The highest mortality in the starved snails was detected in the cypermethrin treatment group, followed by deltamethrin, Fe_2_O_3_ NPs, MgO NPs, and ZnO NPs. Mortality was directly proportional to the NP concentration. All the snails exposed to ZnO NPs survived for 24 h against all four concentrations. In the control group, only one of the snails was dead on the fifth day of the trial, and the rest survived until the end.

**Figure 6:**
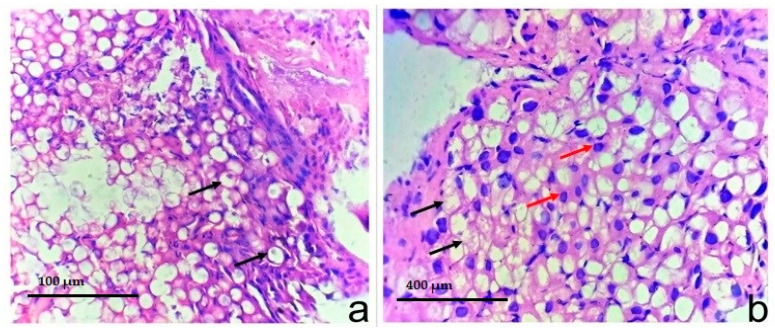
Comparison of normal and nanopesticide-treated digestive glands of snails. (**a**) Control/untreated group (100×): normal secretory cells (black arrows). (**b**) Nanopesticide-treated digestive gland (400×): pyknotic nuclei (red arrows) and vacuolar degeneration (black arrows).

**Histopathology:** Snails exposed to metallic NPs were found to exhibit pathologies at various sites of the digestive glands (Figure 6). The glands were denatured, calcium and excretory cells developed slight vacuolation, and granules were released upon the denaturation of digestive cells. Moreover, basophilic infiltration was evident. Some connective tissue was denatured, and the lumen was widened and later disfigured. Pyknotic nuclei were observed due to the packing of calcium cells with larger calcium spherules. Excretory cells were evident at some sites with debris.

## 4. Discussion

ZnO NPs are among the safest nanomaterials and have been successfully applied in the areas of food, textiles, medicine and healthcare, agriculture, and engineering [28,29,30]. In one previous study of Fe_2_O_3_ NPs, researchers determined that the LC_50_ was 50 µg/mL at 60 min and the LC_99_ was 150 µg/mL at 30 min against *Hyalomma* spp. The authors also showed 85.7% *Hyalomma* mortality under 0 min exposure to 250 µg/mL of Fe_2_O_3_ NPs [31]. In our previous study, neem plant-based ZnO NPs and lemongrass-based ZnO NPs demonstrated LC_50_ values of 4.76 mg/L and 4.92 mg/L and LC_90_ values of 8.87 mg/L and 9.1 mg/L, respectively, against *Hyalomma* ticks [32]. Mortality data from these tests are consistent with other studies of NP-induced toxicity in ticks [33,34]. Previous research has shown that Fe_2_O_3_ NPs and ZnO NPs are safer and more effective NPs than titanium NPs and nickel NPs [33,35] against the larvae of *Hyalomma* ticks. Similarly, the LC_50_ for plant-derived ZnO NPs tested in *Rhichichephalus* ticks was 6.87 mg/L.

It has been proposed that NPs induce toxicity by accelerating or slowing certain cellular mechanisms within host cells [36]. The unique physicochemical surface properties of nanomaterials make them more suitable for downstream functionalization applications [9]. Moreover, ZnO NPs are listed as “generally recognized as safe” by the U.S. Food and Drug Administration (FDA), making them one of the safest NP types in biomedical applications [37]. The safety of ZnO NPs during in vivo testing was demonstrated via the biochemical analysis of subject animal sera [18]. Similarly, in humans, ZnO NPs failed to induce toxicity or bypass the dermal layers 5 days after exposure [30].

MgO NPs have been declared relatively safe materials by the FDA. They are also easy to procure and versatile [38]. Although the exact mechanism by which MgO NPs cause toxicity in insects is still unknown, their anti-pathogenic action has been attributed to the liberation of reactive oxygen species, leading to DNA and (eventual) cell-wall damage due to increased alkalinity [39].

Snails are considered model animals for ecotoxicity assessment of nanomaterials due to their propensity to bioaccumulate and their importance in land and aquatic ecosystems [40]. Snail digestive glands consist of several tubules lined by epithelial cells, digestive cells, calcium cells, excretory cells, thin cells, and digestive tubules. One previous study revealed that exposing snails to ultraviolet-A for 3 h per day for 2 days resulted in digestive lumen widening and disintegration of all cells except calcium cells, while the same exposure over 3 days disintegrated all cells lining the tubules and dramatically increased the number of vacuoles and stained granules [25]. Excessive fluid excretion from the snail’s body may lead to death [41]. Metals have been shown to provoke excretory activity that leads to changes in cell-type composition, expressed as an increase in the relative number or density of basophilic cells. Moreover, cell hypertrophy has also been reported after metal exposure. Therefore, an increase in basophilic cells indirectly indicates digestive cell loss, as these cells are otherwise (under normal conditions) the most abundant. Thus, digestive cells in snails are considered general responders to metal exposure.

## 5. Conclusions

Zinc oxide, magnesium oxide, and iron oxide nanopesticides demonstrated significant anti-tick activity in vitro against *Hyalomma* ticks. Iron oxide demonstrated the lowest lethal concentrations, followed by magnesium oxide and zinc oxide nanopesticides. The results of toxicity assays in snails indicate minimal ecotoxicity compared to commonly used acaricides. Further study is recommended to explore the in vivo effects of applying nanopesticides on tick hosts and in the environment.

## Figures and Tables

**Table 1:** Lethal concentrations owing to application of Fe_2_O_3_, MgO and ZnO nanopesticides against *Hyalomma* ticks.

Acaricide	Tick Stage	LC_50_ (mg/L)	CI	LC_90_ (mg/L)	CI
Iron Oxide	Egg	0.89	0.04–0.92	1.69	0.7–1.9
	Larva	2.83	1.9–3.5	5.58	2.2–5.9
	Adult	4.21	2.7–4.6	8.34	5.3–9.4
Magnesium Oxide	Egg	0.93	0.1–0.93	1.74	1.2–1.9
	Larva	2.91	1.7–3.2	5.77	3.7–6.2
	Adult	4.27	3.6–5.1	8.49	6.4–9.3
Zinc Oxide	Egg	0.96	0.05–0.19	1.80	2.7–3.4
	Larva	3.05	1.1–4.7	5.93	5.1–7.7
	Adult	4.49	3.2–6.2	8.88	6.3–10.1

**Table 2:** Topical application induced toxicity (Mortality%) in snails 24, 72, and 120 h post exposure.

Preparation Name	Concentration (Stock)	Dose per Snail (50 µL)	No. of Snails	Mortality until Day 1 (24 h)		Mortality until Day 3 (72 h)		Mortality until Day 5 (120 h)	
				Ratio	%	Ratio	%	Ratio	%
Cypermethrin (Group 1)	10 mg/mL	500 µg	5	2/5	40	4/5	80	5/5	100
	1 mg/mL	50 µg	5	2/5	40	2/5	40	3/5	60
	0.1 mg/mL	5 µg	5	1/5	20	2/5	40	3/5	60
	0.01 mg/mL	0.5 µg	5	1/5	20	1/5	20	3/5	60
Deltamethrin (Group 2)	10 mg/mL	500 µg	5	1/5	20	3/5	60	4/5	80
	1 mg/mL	50 µg	5	1/5	20	2/5	20	3/5	60
	0.1 mg/mL	5 µg	5	0/5	0	1/5	20	3/5	60
	0.01 mg/mL	0.5 µg	5	0/5	0	1/5	20	2/5	40
MgO (Group 3)	10 mg/mL	500 µg	5	1/5	20	2/5	40	3/5	60
	1 mg/mL	50 µg	5	0/5	0	0/5	0	2/5	40
	0.1 mg/mL	5 µg	5	0/5	0	0/5	0	1/5	20
	0.01 mg/mL	0.5 µg	5	0/5	0	0/5	0	1/5	20
ZnO (Group 4)	10 mg/mL	500 µg	5	0/5	0	1/5	20	3/5	60
	1 mg/mL	50 µg	5	0/5	0	1/5	20	1/5	20
	0.1 mg/mL	5 µg	5	0/5	0	0/5	0	1/5	20
	0.01 mg/mL	0.5 µg	5	0/5	0	0/5	0	0/5	0
Fe_2_O_3_ (Group 5)	10 mg/mL	500 µg	5	1/5	20	2/5	40	4/5	80
	1 mg/mL	50 µg	5	1/5	20	1/5	20	2/5	40
	0.1 mg/mL	5 µg	5	0/5	0	0/5	0	1/5	20
	0.01 mg/mL	0.5 µg	5	0/5	0	0/5	0	0/5	0
Control DMSO (Group 6)	-	50 µL	10	0	0	0	0	1/10	10
Control Distilled water (Group 7)	-	50 µL	10	0	0	0	0	0/10	0

NB: 50 µL was given once directly to the mouth end of snails, in that 500, 50, 5, and 0.5 µg per snail was applied from stock concentrations of 10, 1, 0.1, and 0.01 mg/mL. Percentage mortality on the 1st, 3rd, and 5th day was calculated based on number of dead snails divided by the total number of snails tested in each trial.
