# Correction: Zaheer et al. Acaricidal Potential and Ecotoxicity of Metallic Nano-Pesticides Used Against the Major Life Stages of *Hyalomma* Ticks. *Life* 2022, *12*, 977

**DOI:** 10.3390/life16040660

**Published:** 2026-04-13

**Authors:** Tean Zaheer, Mahmoud Kandeel, Rao Zahid Abbas, Shanza Rauf Khan, Tauseef ur Rehman, Amjad Islam Aqib

**Affiliations:** 1 Department of Parasitology, University of Agriculture, Faisalabad 38040, Pakistan; rao.zahid@uaf.edu.pk; 2 Department of Biomedical Sciences, College of Veterinary Medicine, King Faisal University, Al-Hofuf 31982, Saudi Arabia; 3 Department of Pharmacology, Faculty of Veterinary Medicine, Kafrelshikh University, Kafrelshikh 33516, Egypt; 4 Department of Chemistry, University of Agriculture, Faisalabad 38040, Pakistan; 5 Department of Parasitology, The Islamia University Bahawalpur, Bahawalpur 63100, Pakistan; drtauseef@iub.edu.pk; 6 Department of Medicine, Cholistan University of Veterinary and Animal Sciences, Bahawalpur 63100, Pakistan; amjadislamaqib@cuvas.edu.pk

In order to facilitate readers’ better understanding, some language descriptions and grammar as well as the layout of some chapters have been modified. The authors state that the scientific conclusions are unaffected. This correction was approved by the Academic Editor. The original publication has also been updated [1].

## 1. Text Correction

In Abstract, Line 8–9, “and larval packet tests” should be “and egg immersion tests”. In line 10, “LC_90_” should be “LC_50_”. In line 11, “0.69 mg/L” should be “0.96 mg/L”.

In the Materials and Methods Section, in lines 100–104, “There were seven groups of snails, including two control (negative and positive) and five treatment groups. The seven groups were designated as follows: Group 1 (negative control, distilled water), Group 2 (cypermethrin), Group 3 (deltamethrin), Group 4 (MgO NPs), Group 5 (ZnO NPs), Group 6 (Fe_2_O_3_ NPs), and Group 7 (positive control, DMSO). The five treatment groups (all groups except 1 and 7) were further divided into four sub-groups receiving the following NP concentrations:” should be “There were seven groups of snails, including two control (negative and positive) and five treatment groups. The seven groups were designated as follows: Group 1 (cypermethrin), Group 2 (deltamethrin), Group 3 (MgO NPs), Group 4 (ZnO NPs), Group 5 (Fe_2_O_3_ NPs), Group 6 (positive control, DMSO) and Group 7 (negative control, distilled water). The five treatment groups (all groups except controls) were further divided into four sub-groups receiving the following NP concentrations”.

In the Results Section, the caption of Figure 1, “XRD pattern of synthesized (**a**) Fe_2_O_3_, (**b**) ZnO and (**c**) MgO nanoparticles.” should be “XRD pattern of synthesized (**a**) Fe_2_O_3_, (**b**) ZnO and (**c**) MgO nanoparticles. Intensity has been denoted in ‘a.u’ units”. In lines 170–171, “The egg-laying of Fe_2_O_3_ NP treatment groups was approximately 80%, and the lowest lethal Fe_2_O_3_ NP concentration required to arrest larval hatching was LC_90_ = 1.68 mg/L.” should be “The egg-laying of Fe_2_O_3_ NP treatment groups was 62%, and the lowest lethal Fe_2_O_3_ NP concentration required to arrest larval hatching was LC_90_ = 1.69 mg/L.”. In lines 178–179, “In the control group, only one of the snails was dead on the fifth day of the trial. Most snails died on day 3 of the trial.” should be “In the control group, only one of the snails was dead on the fifth day of the trial, and the rest survived until the end.”.

In the Discussion Section, in line 188, “[29,30]” should be “[28–30]”. In lines 195–196, “than titanium NPs” should be “than titanium NPs and nickel NPs”.

## 2. Error in Figures

According to the Academic Editor’s request, we have replaced Figure 1 with an unedited version. As for Figure 2, we have also improved it by enhancing the existing images and increasing the picture quality.

In the original publication, there were some edits required in Figures 3–5 as published. In Figure 3, detailed pointers and additional stereo-micrographs had been added upon suggestion of the Academic Editor. In Figure 4, the mortality and hatching percentage graphs were simplified upon the Academic Editor’s suggestion. In Figure 5, detailed pointers had been added to enhance clarity upon suggestion of the Academic Editor. The corrected versions of Figure 3, Figure 4 and Figure 5 appear below.

## 3. Error in Table

In the original publication, there was a mistake in Table 1 as published. Units for the lethal concentrations were missing in the table, as previously mentioned in the text. The corrected version of Table 1 appears below.

The authors state that the scientific conclusions are unaffected. This correction was approved by the Academic Editor. The original publication has also been updated.

## Figures and Tables

**Figure 3:**
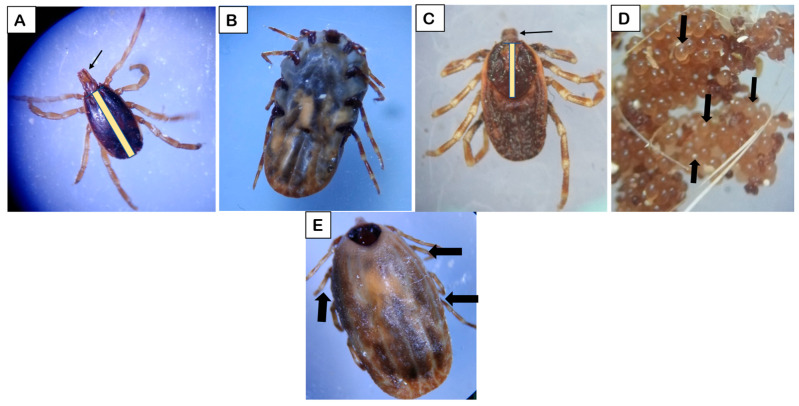
Stereomicrographs of *Hyalomma*. (**A**) male adult–yellow bar: scutum that covers most of the body; arrow: long mouth parts. (**B**) Partially fed female—ventral side showing a smaller degree of engorgement and an increase in size due to blood feeding. (**C**) Un fed female–yellow bar: scutum that covers only 1/3rd of the body; arrow: long mouth part. (**D**) Eggs—egg mass: black arrows indicate freshly laid eggs with a bright appearance. (**E**) Female adult tick showing dorsal view. Black arrows indicate the identification point of *Hyalomma* tick- pale rings on legs.

**Figure 4:**
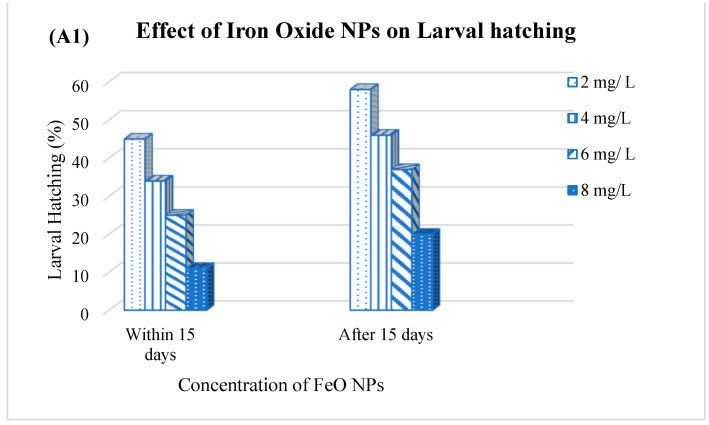

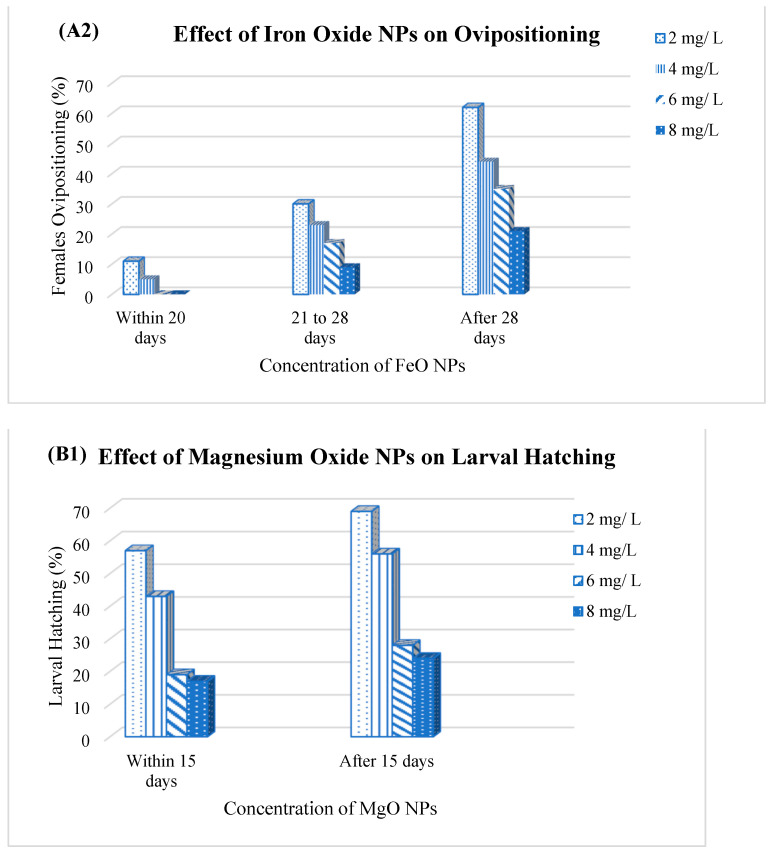

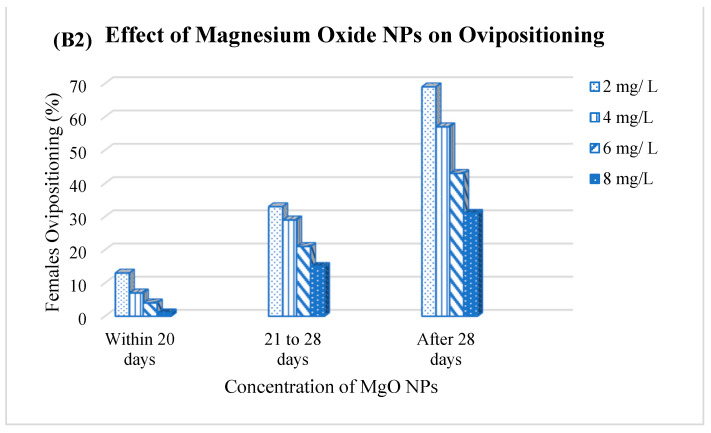

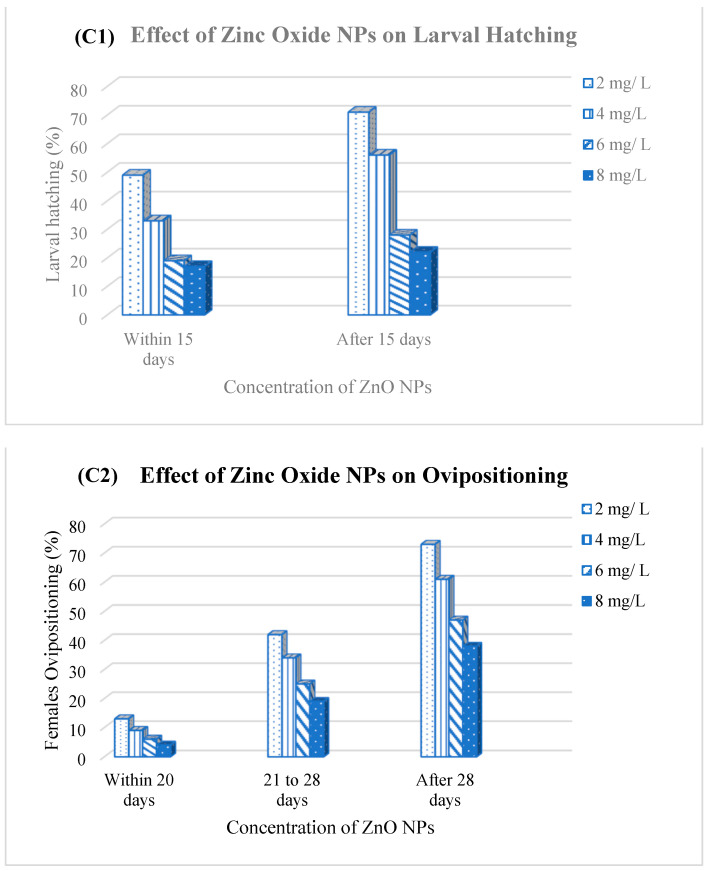
Percentage of larval hatching and adult female oviposition of ticks under the effect of iron oxide (**A1**,**A2**), magnesium oxide (**B1**,**B2**) and zinc oxide nanopesticides (**C1**,**C2**). NPs = nanopesticides/nanoparticles.

**Figure 5:**
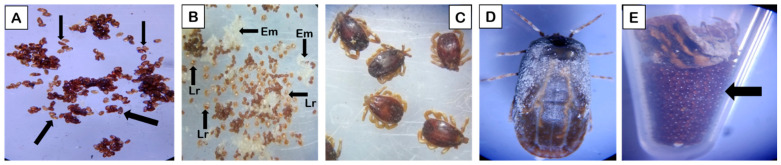
*Hyalomma* ticks subject to nanopesticides. (**A**) Desiccated egg mass with no larval hatching. (**B**) Larval hatching from eggs: small larvae (brown-colored, labelled as ‘Lr’) crawling around white egg masses (labelled as ‘Em’) are visible. (**C**) Dead male ticks. (**D**) Dead female without ovipositioning (dark-colored); no egg mass found, as in control group. (**E**) Ovipositioning in control group, with bright egg masses visible (arrow).

**Table 1:** Lethal concentrations owing to application of Fe_2_O_3_, MgO and ZnO nanopesticides against *Hyalomma* ticks.

Acaricide	Tick Stage	LC_50_ (mg/L)	CI	LC_90_ (mg/L)	CI
Iron Oxide	Egg	0.89	0.04–0.92	1.69	0.7–1.9
	Larva	2.83	1.9–3.5	5.58	2.2–5.9
	Adult	4.21	2.7–4.6	8.34	5.3–9.4
Magnesium Oxide	Egg	0.93	0.1–0.93	1.74	1.2–1.9
	Larva	2.91	1.7–3.2	5.77	3.7–6.2
	Adult	4.27	3.6–5.1	8.49	6.4–9.3
Zinc Oxide	Egg	0.96	0.05–0.19	1.80	2.7–3.4
	Larva	3.05	1.1–4.7	5.93	5.1–7.7
	Adult	4.49	3.2–6.2	8.88	6.3–10.1
